# Multifamily psychoeducation for improvement of mental health among relatives of patients with major depressive disorder lasting more than one year: study protocol for a randomized controlled trial

**DOI:** 10.1186/1745-6215-15-320

**Published:** 2014-08-12

**Authors:** Fujika Katsuki, Hiroshi Takeuchi, Norio Watanabe, Nao Shiraishi, Tohru Maeda, Yosuke Kubota, Masako Suzuki, Atsurou Yamada, Tatsuo Akechi

**Affiliations:** Department of Psychiatric and Mental Health Nursing, Nagoya City University School of Nursing, 1 Kawasumi, Mizuho-cho, Mizuho-ku, Nagoya, 4678601 Japan; Department of Psychiatry and Cognitive-Behavioral Medicine, Nagoya City University Graduate School of Medical Sciences, 1 Kawasumi, Mizuho-cho, Mizuho-ku, Nagoya, 4678601 Japan; Department of Clinical Epidemiology, Translational Medical Center, National Center of Neurology & Psychiatry, 4-1-1 Ogawa-higashi, Kodaira, Tokyo, 1870031 Japan; School of Pharmacy, Kinjo Gakuin University, 2-1723 Omori Moriyama-ku, Nagoya, 4638521 Japan

**Keywords:** Major depressive disorder, Family psychoeducation, Randomized controlled trial

## Abstract

**Background:**

Major depressive disorder (MDD) is a long-lasting disorder with frequent relapses that have significant effects on the patient’s family. Family psychoeducation is recognized as part of the optimal treatment for patients with psychotic disorder. A previous randomized controlled trial has found that family psychoeducation is effective in enhancing the treatment of MDD. Although MDD can easily become a chronic illness, there has been no intervention study on the families of patients with chronic depression. In the present study, we design a randomized controlled trial to examine the effectiveness of family psychoeducation in improving the mental health of relatives of patients with MDD lasting more than one year.

**Methods/Design:**

Participants are patients with MDD lasting more than one year and their relatives. Individually randomized, parallel-group trial design will be employed. Participants will be allocated to one of two treatment conditions: relatives will receive (a) family psychoeducation (four, two-hour biweekly multifamily psychoeducation sessions) plus treatment-as-usual for the patient (consultation by physicians), or (b) counseling for the family (one counseling session from a nurse) plus treatment-as-usual for the patient. The primary outcome measure will be relatives’ mental health as measured by K6 that was developed to screen for DSM-IV depressive and anxiety disorder. Additionally, the severity of depressive symptoms in patients measured by the Beck Depression Inventory–II (BDI-II) scale will be assessed. Data from the intention-to-treat sample will be analyzed 16 weeks after randomization.

**Discussion:**

This is the first study to evaluate the effectiveness of family psychoeducation for relatives of patients with MDD lasting more than one year. If this type of intervention is effective, it could be a new method of rehabilitation for patients with MDD lasting more than one year.

**Trial registration:**

Clinical Trials.gov NCT01734291 (registration date: 18 October 2012).

## Background

Major depressive disorder (MDD) is a common disorder, widely distributed throughout the population, and usually associated with substantial symptoms and role impairment. The prevalence of cases of lifetime MDD was 16.2% in the United States and 12.8% in Europe [1, 2]. The mean duration of a depressive episode was 16 weeks and 59.3% of patients with MDD lasting just one year had severe or very severe role impairment [1]. Moreover, MDD is a long-lasting illness with significant effects on the patient’s family, social life, and work life [3, 4]. Treatment failure results in a low recovery rate and frequent relapses [5]. According to studies on the naturalistic course of MDD, a prospective study in Japan showed that 10 to 20% of patients entering treatment remain chronically depressed without recovery for one or even two years [6]. Once recovered, the cumulative probability of remaining well without subthreshold symptoms was 57% at one year, 47% at two years, and 35% at five years [7].

MDD can also cause severe suffering for family members of the patient in multiple areas including a higher level of divorce [8] and severe financial strain [9]. Fadden *et al*. [10] reported that the burden on the relatives of patients with MDD included restrictions in social and leisure activities, a fall in family income, and a considerable strain on the marital relationship. Relatives of patients with depression found some of the behaviors of the patients to be difficult to bear, and the relatives had negative consequences such as grief, withdrawal, and worrying, which commonly caused problems. However, few relatives know how to deal with the difficult behavior of patients [10]. Moreover, sleep disturbance, odd ideas and/or behavior, and appetite loss in MDD patients were not seen by any relatives as being under the patient’s control, whereas nagging, grumbling, obsessionality, and worrying were seen by a varying proportion of relatives as personality attributes [10]. Therefore, the relatives felt dissatisfaction in that the patient could control those behaviors [10]. Jacob *et al*. [11] reported disruptions in the lives of close family members because of their own worrying and also because of the patient’s lack of interest in things and feelings of worthlessness. Taken together, these findings suggest that living with a patient with MDD is a source of strain and emotional distress for relatives.

Among relatives of patients with MDD, the patient’s behavior and mood disturbance and relative’s emotional distress were associated with the relatives’ mental health [12–14]. The difficultness of maintaining functioning family relationships (for example the patient may have marital problems, poorer communication, and no problem-solving skills) was associated with poorer long-term outcome for the depression [15, 16]. Rounsaville *et al*. [17] reported that a reduction in the number of marital disputes was associated with improved depressive symptoms and social functioning after eight months of individual psychotherapy in depressed female outpatients. Several studies reported that the quality of family functioning was associated with the relapse rate. The family’s expressed emotion (EE) is a good predictor of whether a patient relapses; Hooley *et al*. [18] reported that 59% of patients whose spouses had high levels of EE relapsed, although no patients living with low-EE spouses did so over a nine-month follow-up period. Although Hayhurst *et al*. [19] reported that there was no clear association between the EE of a spouse and the recurrence of depression in the patient, five studies reported that a high EE predicted a high relapse rate [18, 20–23].

These studies suggest the need for a more family-oriented approach in the treatment of MDD. However, a review conducted by Henken *et al*. [24] reported that family therapy such as behavioral intervention including psychoeducation, psychodynamic intervention, and systemic intervention for the families of patients with depression, seems to be more effective than no treatment or being placed on a waiting list. However it remains unclear how effective this intervention is in comparison with other interventions such as group intervention, individual cognitive intervention, and behavioral intervention. In spite of the lack of high-quality evidence in this field, family therapy is already a widely-used intervention for the treatment of depression [24].

Family psychoeducation is recognized as part of the optimal treatment for patients with a psychotic disorder [25, 26]. This intervention has been shown to reduce the rates of relapse and hospitalization among individuals with psychotic disorders and is recognized as an evidenced-based treatment for psychotic disorders [27]. Two randomized controlled trials have found that family psychoeducation is effective in enhancing the course of MDD [28, 29]. In a study of adolescents with MDD, patients in the group who received family psychoeducation showed greater improvements in social functioning and adolescent-parent relationship than the control group [28]. Among patients with MDD in partial or full remission, patients who were treated with the family psychoeducation had a significantly lower relapse rate than patients who were in the control group [29]. However, neither of these two trials assessed relatives’ mental health as the primary outcome. Additionally, although MDD can easily become chronic, there has been no intervention study for the families of patients with MDD lasting more than one year.

### Aims

In the present study, we perform a randomized controlled trial to examine the effectiveness of family psychoeducation in improving the mental health of the relatives of patients with MDD lasting more than one year. The hypothesis is that, compared with relatives who receive one regular counseling session from a nurse, relatives receiving family psychoeducation will show a greater improvement in mental health as measured by K6 scale at 16 weeks post-randomization.

## Methods/Design

### Design overview

**Figure 1:**
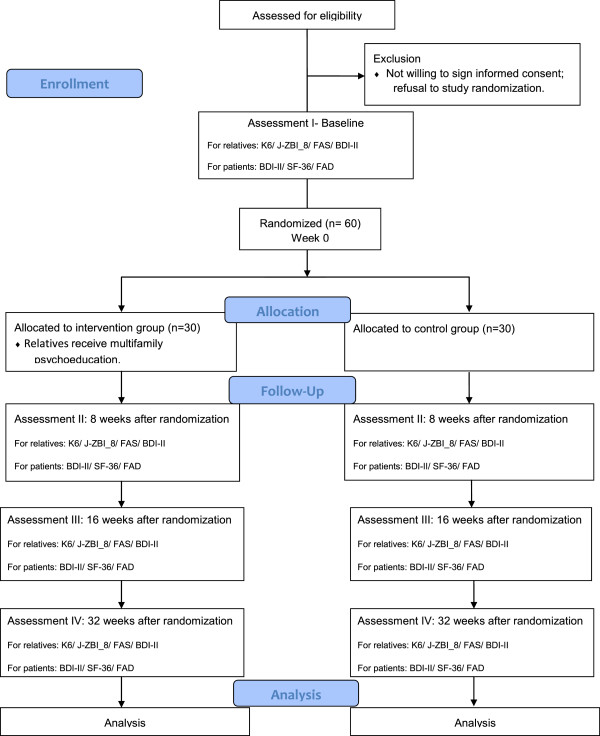
**Participant flow diagram.** K6, J-ZBI_8; The Japanese version of the Zarit Burden Interview short version, FAS; The Japanese version of the Family Attitude Scale, BDI-II; Beck Depression Inventory-II, SF-36; The Medical Outcomes Study 36-item short form health survey (SF-36) version 2, FAD; The Japanese version of the Family Assessment Device.

This randomized controlled trial will be conducted in patients with MDD who will be allocated to one of two arms: family psychoeducation in addition to treatment-as-usual for the patients, and treatment-as-usual. Treatment-as-usual consists of consultation administered by a physician and counseling by a nurse. The primary endpoint is improvement of the mental health of patients’ relatives as measured by K6 scale at 16 weeks (Figure 1). We defined mental health as the state of health of the mind and that when the mental health of relatives was in an unhealthy state, the person suffered from mental disorder such as depressive and anxiety disorder.

### Participants: inclusion and exclusion criteria

The target population will be patients with MDD lasting more than one year and their relatives (fathers, mothers, husbands, wives, daughters, and sons of patients). Inclusion criteria will be: the patient meets the criteria for MDD according to the DSM-IV (Diagnostic and Statistical Manual of Mental Disorder 4^th^ edn) based on the consensus rating by psychiatrists (who have more than five years’ experience as a physician and more than two years’ experience as a psychiatrist) in charge without using a structured interview; the patient receives antidepressant therapy; the patient had the first episode of MDD more than one year prior; the patient currently fulfills the diagnostic threshold for major depressive episode or partial remission; the patient and their family member(s) are aged between 18 and 85 years; the patient lives with his or her family at the time of participating in this study and is expected to live with his or her family during the investigation period. Exclusion criteria will be: patients who undergo electroconvulsive therapy (ECT) during the investigation period and patients who are at serious suicidal risk.

### Procedure

This study was approved by the Ethics Review Committee of Nagoya City University Graduate School of Medicine (reference number 679). All participants will provide written informed consent after the purpose and procedures of the study are explained. This study is registered at ClinicalTrials.gov under number NCT01734291. We will provide eligible patients with an ID number and then ask patients and their relatives to provide informed consent and complete a baseline assessment (Assessment I). After providing informed consent and completing the baseline assessment, participants will be randomized. Assessment will occur at baseline, before randomization, and at 8, 16, and 32.

### Randomization

Participants will be randomly allocated to one of the two groups with equal probability. This random assignment will be made in a 1:1 ratio. An independent statistician will generate the random allocation sequences by a computer using minimization [30], and stratify the relatives according to the severity of mental state (K6 score of 5 or more, or less than 5). Allocation sequences will be kept centrally and the allocation will be provided by facsimile to us. The randomization schedule is not available to anyone except the statistician.

### Treatment

#### Multifamily psychoeducation

The family intervention program, which we call ‘multifamily psychoeducation’, is based on the McFarlane Model [31], the Evidence-Based Practices Toolkit for Family Psycho-Education [32], and the standard model of the Japanese Network of Psychoeducation and Family Support Program (JNPF) [33]. The multifamily psychoeducational program will consist of four sessions. Each of the four multifamily psychoeducational program groups will consist of the relatives of approximately four patients. The staff will consist of one or two psychiatrists, one or two nurses, one pharmacologist, and one social worker. The teaching materials for the relatives of the patients are a videotape produced by the Department of Neuropsychiatry, Kochi Medical School [34], including the videotaped interview of the experience of a patient with MDD, an explanation of the cause of MDD using computer graphics images of synapses and neurotransmitters, and a booklet developed by our department. At the first session we will give the participants information on the causes and symptoms of major depression, at the second session we will provide information on drug treatment, at the third session we will provide information on community resources, and at the fourth session we will provide guidelines for families caring for patients. After the lecture in each session, we will provide supportive group therapy focusing on problem-solving skills for approximately 90 minutes. In the group therapy sessions, the participants will be encouraged to give a narrative of their subjective experience in taking care of the MDD patient. Each session will last approximately two hours. The groups will meet once every two weeks over the course of six weeks.

#### Control group

The patients in both the intervention and control groups will receive standard outpatient or inpatient treatment administered by physicians. Outpatient treatment consists of evaluation of psychiatric symptoms, antidepressant pharmacotherapy, and supportive psychotherapy on a bi-weekly or-four-weekly basis. Inpatient treatment consists of sufficient rest for the patient, evaluation of psychiatric symptoms, antidepressant pharmacotherapy, and supportive psychotherapy. All of the participants will receive some case management. Family treatment in the control group consists of one counseling session administered by a nurse. This counseling consists of listening to any issues or problems and providing any information that is ask for. The information requested is usually regarding their communication, relapses, and how to take their drugs. We selected this counseling treatment as the control group’s treatment, because active listening to relatives’ suffering and giving information on recuperation from nurses are within the scope of treatment-as-usual. The one session of counseling by a nurse will last 45 minutes.

#### Therapist training, supervision and fidelity control

Authors FK, NS, and MS were each trained and certified as a family psychoeducation instructor by the JNPF [33]. All staff except one pharmacologist had participated in intensive training which consisted of more than eleven hours using the treatment manual of the JNPF [33]. In order to ensure the fidelity of each session, all sessions will be audiotaped and 25% of each condition will be randomly selected, evaluated, and commented about for improvements continuously by an independent researcher. The counseling session in treatment-as-usual administered by a nurse will also be audiotaped and 20% of each condition will be randomly selected and evaluated by an independent researcher. The raters will each be trained and certified as a family psychoeducation instructor by the JNPF.

### Outcome measure

#### Primary outcome measure of family members - K6

The K6 questionnaire is a six-item self-report questionnaire that was developed to screen for any DSM-IV depressive and anxiety disorders within 30 days prior to its administration and which can also be used to quantify nonspecific psychological distress in general [35]. The participants of this study will not be individuals with a mental disease but will be among the general population, and our previous study showed that the K6 was excellent for measuring changes in the mental health of relatives of MDD patients [36]. K6 has a high screening performance for mood and anxiety disorder, equal to that of the Center for Epidemiologic Studies-Depression Scale (CES-D) [37]. Each item in the K6 questionnaire is rated between 0 = ‘none of the time’ and 4 = ‘all of the time’, and the total score therefore ranges from 0 to 24. Two independent validation studies found the K6 to have an area under the receiver operating characteristic curve between 0.86 and 0.89 in predicting the diagnosis of mental illness based on comprehensive diagnostic interviews [35, 38]. The Japanese version of the K6 questionnaire showed excellent efficacy in screening for anxiety and mood disorders in the Japanese general population, with an area under the receiver operating characteristic curve of 0.94 [39]. The Japanese version of the K10 questionnaire also showed excellent efficacy in screening for anxiety and mood disorders in the Japanese general population, with an area under the receiver operating characteristic curve of 0.94 [39]. In this study, we will use the K6 as it has fewer items than the K10.

#### Secondary outcomes of family members

##### The Japanese version of the Zarit Burden Interview short version (J-ZBI_8)

The Zarit Burden Interview is widely used to assess caregiver burden [40]. The Japanese version of the Zarit Burden Interview (J-ZBI) was developed by Arai *et al*., and the eight-item short version of the J-ZBI (J-ZBI_8) was also developed by Arai *et al*. [41–43]. The items in the J-ZBI_8 are rated on a five-point Likert scale (0 = never to 4 = very often) and the scores on the J-ZBI_8 range from 0 to 32. Cronbach’s α of the J-ZBI_8 was 0.89, and the Pearson’s correlation coefficient between scores on the J-ZBI and J-ZBI_8 was 0.93 [41].

##### The Japanese version of the Family Attitude Scale (FAS)

The FAS, developed by Kavanagh *et al*. [44], is a 30-item self-report instrument and measures families’ EE. The scores for each item are added to give a total score that ranges from 0 to 120, with higher scores indicating higher levels of burden or criticism [44]. A higher FAS rating was significantly correlated with higher levels of criticism (r = 0.44), hostility (r = 0.41), and emotional overinvolvement (EOI) (r = 0.27) in the Camberwell Family Interview (CFI) [45]. The Japanese version of the FAS, developed by Fujita *et al*. [46], showed excellent validity. The relative sensitivity and specificity of EE assessment with the FAS compared with the criticism component of the CFI were 100% and 88.5%, respectively [46].

##### Beck Depression Inventory-II (BDI-II)

BDI-II is a 21-item self-report instrument to assess the existence and severity of symptoms of depression [47]. It has good psychometric properties and acceptability. Each item is rated on a four-point scale ranging from 0 to 3. Studies consistently support the BDI-II as a reliable, internally consistent, and valid scale for assessing depression in psychiatric outpatients and the general population, and in primary care settings [47–49]. The reliability and validity of the Japanese version have been found to be excellent [50].

#### Secondary outcomes in patients

##### Beck depression inventory-II (BDI-II)

We also selected BDI-II as an outcome measure to evaluate the severity of patients’ depressive symptoms.

##### The Medical Outcomes Study 36-item short form health survey (SF-36) version 2

The SF-36 is a self-report questionnaire to assess general quality of life. It contains 36 items that constitute eight measures of physical functioning (PF), role physical (RP), bodily pain (BP), social functioning (SF), general health perceptions (GH), vitality (VT), and mental health (MH). It also provides two summary measures, the physical component summary (PCS) and the mental component summary (MCS). The PCS is associated with PF, RP, BP, GH, and VT. The MCS is associated with MH, RE, SF, VT, and GH. The score of each measure ranges from 0 to 100, and the higher the score, the higher the quality of life. The Japanese version has shown good validity in the general population of Japan [51, 52].

##### The Japanese version of the Family Assessment Device (FAD): J-FAD

The FAD is a 60-item self-report questionnaire developed by Epstein *et al*. [53] to assess the six dimensions of the McMaster Model of Family Functioning as well as the family’s overall level of functioning. The FAD consists of seven subscales: problem solving, communication, roles, affective responsiveness, affective involvement, behavior control, and general functioning. The Japanese version of the FAD was developed by Saeki *et al*. and has shown good validity [54].

### Sample size and statistical power

Sample size was based on a power analysis conducted for the K6 score. Effect sizes were estimated from our previous pilot study [36] (the mean change in the K6 scores pretreatment to post-treatment was 4.9 in 32 relatives of patients with MDD). The change in the K6 scores pretreatment to post-treatment (16 weeks after the randomization) was 4.5 ± 2.5 (mean ± SD) in the family psychoeducation group and 2 ± 2.5 in the control group. With a power of 0.9 to detect a significant difference at *P* = 0.05 (two-sided), it was calculated that 23 patients would be required for each arm. Thus, allowing for a 20% dropout rate, 30 participants would need to be recruited per group.

### Statistical analysis

Statistical analyses will be performed using SPSS 19 J for Windows. Descriptive data analysis will be conducted by calculating mean scores and standard deviation. All analyses will be based on the intent-to-treat model. We will use analysis of covariance at 16 weeks if there is no missing data at that time. If missing data is observed, we will use the maximum likelihood mixed model which accounts for missing data, provided that the data are missing at random, conditional on the covariates and the baseline values of the outcome. *P* <0.05 will be set to test the null hypothesis.

## Discussion

One randomized controlled trial found that family psychoeducation is effective in enhancing the course of MDD [29]. MDD can easily become a chronic condition, therefore, it is reasonable to assume that relatives of these patients with MDD experience a heavy psychosocial burden and show increased rates of depression and anxiety [55, 56]. However, there has been no intervention study on the mental health of families of patients with chronic depression. With such a background, this will be the world’s first study on family psychoeducation in families of patients with MDD. Moreover, the results will provide useful suggestions for the comprehensive treatment of chronic depression.

This study has four major strengths. First, since a variety of workers such as physicians, nurses, pharmacologists, and psychiatric social workers participate in this family psychoeducational program, it is possible to assess the family from various points of view. Physicians are specialists of psychiatric treatment (especially pharmacotherapy); nurses are experts in providing guidance on everyday life to patients and their relatives; the pharmacologist is an expert in the effects of medicine; and the psychiatric social worker is knowledgeable about community resources. Thus, information is provided on a wide range of topics from disease to treatment to communication, and each expert is in charge of providing information.

Second, the method of family psychoeducation employed in this study is group therapy that improved the McFarlane model according to the Japanese Network of Psychoeducation and Family Support Program and focuses more on the strength of the family. With regard to the structure of the multifamily group sessions, common steps in the problem-solving process shared by the standard model of the Japanese Network of Psychoeducation and Family Support Program and the McFarlane Model are: (1) socializing with other families, (2) defining the problem or goal, (3) listing all possible solutions suggested by the group members, and (4) the family member who presented the problem chooses the solution that best fits the situation. However, the standard model of the Japanese Network of Psychoeducation and Family Support Program differs from the McFarlane Model with regard to several points. In the standard model of the Japanese Network of Psychoeducation and Family Support Program, the advantages and disadvantages of each solution are not discussed in detail and an action plan to carry out the solution is not formed. According to the model, it is easier for all members in the group to talk about and share their experiences at each session and give advice to each other instead of focusing on the solution of a problem of only one member in the group.

Third, the primary outcome of the study is the mental health of the families, but the outcome of the patients is also evaluated. In addition, not only the family but also the patient is evaluated from a variety of aspects, including family function and depressive symptoms. Fourth, since the sample size was calculated in this study based on the pilot study we had previously carried out [54], it is possible to accurately estimate the required number of cases.

This study has some limitations. First, the diagnosis of MDD will not be made by a structured clinical interview and comorbidities such as anxiety disorder are included. However, it is not common to have a structured interview for diagnosis in daily clinical practice and it is known that MDD has a number of comorbidities. Second, an objective evaluation tool such as the Hamilton Depression Rating Scale will not be used, but the self-report inventory will be used for evaluation of patients’ symptoms. Third, families with low levels of stress (K6 scores of four points or less) will be included among the subjects in the study. In daily clinical practice, patients will not be excluded from receiving care just because they have low levels of stress according to the questionnaire for the family. Finally, although an attention-placebo arm must be employed originally, we aim to conduct the study to examine the effectiveness of family psychoeducation for improvement of the mental health of relatives of patients with MDD, including eight-hour contact with medical professionals, but not to examine the efficacy of family psychoeducation for MDD itself. Taken together, it would be reasonable to say that this is an effectiveness study under conditions more resembling an actual clinical setting and the results would be widely applicable to clinical practice immediately. Rehabilitation elements are important in recovery from chronic disease, but no comprehensive rehabilitation program for chronic MDD has been sufficiently developed. If the efficacy is confirmed in this study, it will be one of the useful rehabilitation programs for chronic MDD.

## Trial status

The trial is currently in the recruitment phase. The first group was randomized on 12 October 2012.

## Abbreviations

BDI-II: Beck depression inventory
CES-D: Center for epidemiologic studies- depression scale
ECT: Electroconvulsive therapy
EE: Expressed emotion
FAS: the Japanese version of the Family Attitude Scale
J-FAD: the Japanese version of the Family Assessment Device
JNPF: the Japanese Network of Psychoeducation and Family Support Program
J-ZBI_8: the Japanese version of the Zarit Burden Interview short version
MDD: Major depressive disorder
SF-36: the MOS 36-item Short Form Health Survey version 2.
